# Effect of Obstructive Sleep Apnea Syndrome on Macular Edema Severity and Response to Intravitreal Ranibizumab in Diabetic Retinopathy and Retinal Vein Occlusion

**DOI:** 10.7759/cureus.81385

**Published:** 2025-03-28

**Authors:** Lagnajeeta Banerjee, Priti Singh, Abhishek Goyal, Vidhya Verma, Saroj Gupta, Samendra Karkhur

**Affiliations:** 1 Ophthalmology, All India Institute of Medical Sciences, Bhopal, Bhopal, IND; 2 Pulmonary and Critical Care Medicine, All India Institute of Medical Sciences, Bhopal, Bhopal, IND; 3 Respiratory Medicine, Graphic Era Institute of Medical Sciences, Dehradun, IND

**Keywords:** anti-vegf, central retinal vein occlusion (crvo), diabetic macular edema, diabetic retinopathy, intravitreal injection, macular edema, obstructive sleep apnea syndrome (osas), retinal vein occlusion, retina vascular occlusion, sleep study parameters

## Abstract

Background

Obstructive sleep apnea syndrome (OSAS) is a condition characterized by repeated episodes of partial or complete blockage of the upper airway during sleep, leading to disrupted breathing and poor sleep quality. In individuals with OSAS, blood oxygen levels can drop significantly due to repeated interruptions in breathing during sleep. The retina is highly sensitive to oxygen levels, and prolonged hypoxemia can contribute to retinal damage and worsen macular edema.

Objective

The study aims to correlate the effect of OSAS on the severity of macular edema as evaluated through optical coherence tomography (OCT) in patients with diabetic retinopathy and retinal vein occlusion. Additionally, the study seeks to assess treatment outcomes following anti-vascular endothelial growth factor (VEGF) therapy. It will also evaluate the relationship between subfoveal choroidal thickness (SFCT) and OSAS using enhanced depth imaging OCT.

Methods

Patients with macular edema secondary to diabetic retinopathy or retinal vein occlusion were identified through a complete ophthalmological examination and referred to pulmonology for level 1 polysomnography to diagnose concomitant OSAS. All patients received anti-VEGF ranibizumab for macular edema, with monthly follow-ups until the macular edema resolved (central macular thickness (CMT) less than 250 µm) or until three intravitreal ranibizumab injections were administered. All data were collected and analyzed.

Results

The study included 12 participants, with a mean age of 51.33 ± 14.16 years, a mean height of 163.41 ± 7.21 cm, and a mean weight of 65.08 ± 13.31 kg. Among the participants, 25% had mild OSAS, 50% had moderate OSAS, and 25% had severe OSAS. All participants showed improvement in visual acuity when comparing the first and third visits, with a p-value < 0.05 for the mild and moderate OSAS groups. The decline in CMT post-intravitreal ranibizumab was significant across all levels of OSAS, with a p-value < 0.05. The mean SFCT was reduced in all participants relative to normal age-matched controls; however, these results were not statistically significant. A weak positive correlation was observed between the apnea-hypopnea index and CMT, as well as between the oxygen desaturation index and CMT.

Conclusions

The study findings indicate that while there was no significant association between the severity of OSAS and CMT, there was a statistically significant improvement in visual acuity following anti-VEGF injections in patients with mild to moderate OSAS. In cases of severe OSAS, the improvement in vision was minimal and statistically insignificant. Additionally, the mean SFCT was significantly reduced in the study population compared to normal age-matched controls, but no significant differences were found when comparing the different severities of OSAS individually.

## Introduction

Macular edema is the accumulation of fluid in the outer plexiform layer and/or the inner nuclear layer, leading to an increase in central retinal thickness. It is one of the leading causes of visual loss. The macula is particularly susceptible to edema due to the high rate of fluid production associated with densely packed cone photoreceptors and its low rate of extracellular fluid resorption, primarily because of the presence of a central avascular zone. Additionally, Henle’s fiber layer arrangement turns the macula into a potential reservoir for fluid. Macular edema can result from various intraocular and systemic diseases, often leading to significant vision impairment. Notably, diabetic macular edema is a major cause of vision loss, affecting both type 1 and type 2 diabetic patients. Additionally, macular edema caused by retinal vein occlusion results in a significant disease burden in the population.

In diabetes mellitus, chronic hyperglycemia releases inflammatory cytokines, including IL-6, IL-8, and IL-1β, and growth factors like TNF-alpha and matrix metalloproteinases. These factors lead to the accumulation of advanced glycation end products and the blood-retinal barrier disruption. Increased retinal vascular permeability subsequently causes hypoxia and upregulation of VEGF signaling pathways, accumulating interstitial fluid and, hence, macular edema [1].

Retinal vein occlusion leads to increased venous and capillary back pressure, causing stagnation of blood flow, damage to surrounding endothelial cells, thrombogenesis, and potential occlusion. This situation markedly increases interstitial fluid levels and decreases capillary perfusion, leading to global or localized retinal ischemia, depending on the location of the occluded vein. The vascular endothelial growth factor (VEGF) release contributes to excessive vascular permeability, resulting in macular edema [2].

The severity of macular edema is influenced by various factors, one of which is the presence of obstructive sleep apnea syndrome (OSAS). OSAS is a sleep disorder characterized by repeated episodes where breathing stops or significantly reduces during sleep due to a complete or partial airway collapse despite the effort to breathe. This interruption in airflow leads to intermittent hypoxia and fragmented sleep, resulting in disturbed sleep architecture, sympathetic activation, and elevated inflammatory markers in the blood. Consequently, this can cause increased daytime blood pressure, endothelial damage, oxidative stress, and the release of VEGF, which accelerates retinal vascular damage [3]. Additionally, the cycles of hypoxia and arousal associated with OSAS can induce structural changes in the choroidal vasculature, decreasing choroidal thickness.

Diagnosis of OSAS is typically performed through an overnight polysomnography (PSG) test. This comprehensive evaluation records electroencephalogram waves, oxygen saturation, heart rate, breathing patterns, and eye and leg movements to identify sleep disorders and assess their severity. Early detection of macular edema is crucial for accurate diagnosis and effective management. The introduction of optical coherence tomography (OCT) has made it an essential tool in diagnosing and treating macular edema.

Macular edema is traditionally managed via a stepwise therapeutic approach, with strict control of underlying systemic conditions being paramount. In recent years, updated treatment methods have emerged, focusing on intravitreal corticosteroid and anti-VEGF injections. Limited studies examine how OSAS affects the severity of macular edema and treatment responses. Research indicates that patients with macular edema who also suffer from OSAS tend to have a poorer response to anti-VEGF therapy; however, this area has not been extensively explored. This study emphasizes the importance of diagnosing OSAS in managing macular edema resulting from diabetic retinopathy and retinal vein occlusion.

## Materials and methods

This is a single-center, prospective longitudinal study conducted over a period of 18 months in the Department of Ophthalmology at All India Institute of Medical Sciences (AIIMS), Bhopal, enrolling participants diagnosed with macular edema secondary to type 2 diabetes mellitus or retinal vein occlusion; all consented to undergo overnight PSG (level 1 study) from September 2021 through March 2023. All patients with macular edema who met the inclusion criteria were enrolled after written informed consent. The study adhered to the tenets of the Declaration of Helsinki and received approval from the Institutional Human Ethics Committee of AIIMS Bhopal. The inclusion criteria for study participants included adults over 18 years of age with macular edema secondary to diabetes mellitus or retinal vein occlusions, diagnosed with OSAS through overnight level 1 PSG, and willing to comply with therapy and follow-up. Patients who were unwilling to participate in the study, individuals under 18 years of age, and those with other causes of macular edema (such as posterior uveitis, vitreomacular traction, retinal dystrophies, etc.) were excluded. Other macular conditions such as age-related macular degeneration, heredomacular degeneration, or macular scarring were also excluded. Additionally, patients with conditions like proliferative diabetic retinopathy (PDR), vitreous hemorrhage, or tractional retinal detachment were not included. Furthermore, individuals who had undergone any ocular surgery or retinal laser treatment, including focal or grid macular laser treatment, steroid injections into the posterior segment within the last three months, or those with incomplete medical records were also excluded from the study.

Spectral domain OCT with enhanced depth imaging (EDI) of the retinal area centered on the fovea was performed using the Cirrus HD-OCT (Carl Zeiss Meditec AG, Jena, Germany). This involved an optical source of Super Luminescent Diode at 840 nm, with a scan speed of 27-68K A-scans per second. The A-scan depth was 2.0 mm in tissue, with an axial resolution of 5 μm and a transverse resolution of 15 μm in tissue. EDI mode provided by the manufacturer was used to obtain images of the choroid. Transfoveal horizontal and vertical line sections, consisting of 100 averaged scans, were acquired to measure the subfoveal choroidal thickness (SFCT). Central subfield thickness from the internal limiting membrane to the retinal pigment epithelium (RPE) was measured using internal calipers from the Cirrus HD-OCT 500 within the 6 mm macular map. SFCT was measured using built-in calipers from the outer portion of the RPE to the scleral-choroid interface.

According to the American Academy of Sleep Medicine (AASM) guidelines [4], at least one of the following criteria must be present for diagnosing OSAS: the patient reports daytime sleepiness, unrefreshing sleep, fatigue, insomnia, and/or unintentional sleep episodes during wakefulness; the patient awakens with breath-holding, gasping, or choking; the patient’s bed partner reports loud snoring or breathing interruptions, or both, during the patient’s sleep; PSG shows more than five scoreable respiratory events per hour of sleep and/or evidence of respiratory effort during each event; PSG shows more than 15 scoreable respiratory events per hour of sleep and/or evidence of respiratory effort during each event; and other current sleep disorders, medical or neurological disorders, medication use, or substance use do not better explain the patient’s condition.

All patients diagnosed with macular edema secondary to diabetic retinopathy or retinal vein occlusion were referred to the Pulmonary Medicine Sleep Laboratory at AIIMS, Bhopal. Patients underwent level III PSG using the Philips Alice One (Philips Respironics, Murrysville, Pennsylvania, USA), with thoracic movements measured and the application of a nasal cannula and saturation probe. Patients with an apnea-hypopnea index (AHI) greater than 15 underwent a split-night study (level I PSG) with manual CPAP titration to determine optimal pressure. Level I PSG was conducted at the Alice 6 PSG laboratory (Philips Respironics). Electroencephalography, chin electromyography, leg electromyography, electrooculography, oxygen saturation measurements, and chest and abdominal movements were monitored with all patients’ z-RIP belts and body sensors.

The AASM criteria [4] for scoring OSAS were utilized. Apnea was defined as the cessation of airflow through the nose for more than 10 seconds, and hypopnea was defined as a reduction in airflow by ≥30%, associated with a decrease in pulse oximetry reading (desaturation) by ≥3%. The severity of OSAS was described by the number of apnea and hypopnea episodes per hour of sleep, measured by the AHI. OSAS was defined as AHI ≥5 and categorized into three severity grades based on AHI: mild-moderate (AHI 5-29.9) and severe (AHI ≥30). All patients with AHI ≥15 were titrated with CPAP, following the AASM scoring rules for CPAP titration from 2008, as described by Richard Berry. Statistical analysis was performed using IBM SPSS Statistics for Windows, Version 23.0 (Released 2015; IBM Corp., Armonk, NY, USA). Continuous and categorical variables were expressed as mean ± SD and percentages. Two-sided p-values were considered statistically significant at p < 0.05. Descriptive statistics were compiled for age, height, weight, BMI, gender, and comorbidity. A repeated measures ANOVA test was used to see changes in visual acuity, central macular thickness (CMT), and SFCT in mild/moderate/severe OSAS. The chi-square test was used to see an association between mild/moderate/severe OSAS and the number of injections. Pearson’s correlation was used to see a correlation of AHI, oxygen desaturation index, and time under 90% oxygen saturation (T90) with CMT in the first visit, second visit, and third visit. The visits were one month apart, and the total number of visits depended upon the number of injections required for the edema to resolve completely, which could range from a minimum of one injection to a maximum of three injections. PSG was conducted only once at the beginning of the study. The protocol followed for the anti-VEGF therapy in our study was initial monthly loading doses, one month apart, followed by PRN dosing.

Regarding the anti-VEGF treatment, an injection of a 2.3 ml vial of ranibizumab (10 mg/0.1 ml) was used, and the dose of 0.05 ml was injected per eye as per the study protocol. The preparation used was a generic formulation named Razumab by Intas Pharmaceuticals Ltd. (Ahmedabad, Gujarat, India) across the study since it was available as a hospital supply drug at the study site.

## Results

In this prospective longitudinal study, 12 participants were enrolled. Most participants were over 50, with a mean age of 51.33 ± 14.16 years. The average BMI of the participants was 24.24 ± 4.04. Among the participants, nine were males, representing 75% of the study group, while three were females, accounting for 25%. Both eyes of all participants were examined during visits held every three months. Two patients had type 2 diabetes mellitus, six patients had hypertension, two had both diabetes and hypertension, one patient had hyperhomocysteinemia, and one did not have any associated systemic comorbidities. Three patients were diagnosed with a mild degree of OSAS, six with a moderate degree of OSAS, and the remaining three patients had a severe degree of OSAS, as evaluated through overnight PSG.

The mean visual acuity, measured on a logarithmic scale for mild OSAS cases, was 1.00 ± 0.40 during the first visit, improving to 0.20 ± 0.34 by the third visit. In cases of moderate OSAS, the mean visual acuity started at 0.76 ± 0.15 during the first visit and improved to 0.23 ± 0.08 by the third visit. For severe OSAS, the mean visual acuity was recorded at 0.73 ± 0.30 during the first visit and improved to 0.33 ± 0.23 during the third visit (Table 1).

**Table 1:** Improvement in visual acuity among patients with various degrees of OSAS OSAS, obstructive sleep apnea syndrome

Obstructive sleep apnea	Visual acuity	Mean	SD
Mild (N = 3)	First visit	1.00	0.40
	Second visit	0.56	0.40
	Third visit	0.2	0.34
Moderate (N = 6)	First visit	0.76	0.15
	Second visit	0.46	0.20
	Third visit	0.23	0.08
Severe (N = 3)	First visit	0.73	0.30
	Second visit	0.50	0.26
	Third visit	0.33	0.23

The greatest improvement in visual acuity was observed in patients with mild OSAS, followed by those with moderate OSAS. In contrast, the least improvement was seen in patients diagnosed with severe OSAS (Table 2).

**Table 2:** Change in visual acuity between presentation and final follow-up visit * and ** denote statistical significance (p ≤ 0.05).

Obstructive sleep apnea	Visual acuity		Mean difference	p-Value
	First visit	Last visit		
Mild (N = 3)	1.00 ± 0.4	0.20 ± 0.34	0.8	0.02*
Moderate (N = 6)	0.76 ± 0.15	0.23 ± 0.08	0.53	<0.001**
Severe (N = 3)	0.73 ± 0.30	0.33 ± 0.23	0.4	0.076

A significant change in CMT was noted across all degrees of OSAS, with the mild OSAS group showing the most substantial reduction in CMT between the first and third visits compared to the moderate and severe groups (Table 3). This observation provides a greater understanding of the role of hypoxia in affecting the outcomes of an ischemic phenomenon such as diabetic macular edema and the macular edema associated with retinal vein occlusions. The presenting visual acuity and CMT were not considered since these values may be affected by the duration between the onset of signs and symptoms and when participants sought medical attention. The change in these values provided a more objective assessment of the underlying factors under study influencing this change.

**Table 3:** Change in CMT through visits * and ** denote statistical significance (p ≤ 0.05). CMT, central macular thickness

Obstructive sleep apnea	CMT		Mean difference	p-Value
	First visit	Last visit		
Mild (N = 3)	806.33 ± 192.64	358.33 ± 107.9	448	0.018*
Moderate (N = 6)	611.50 ± 167.25	254.0 ± 40.65	357.5	0.002**
Severe (N = 3)	614.66 ± 44.63	343.66 ± 108.39	271	0.022*

The improvement in visual acuity, directly related to the severity of macular edema, is most significant in participants with mild OSAS compared to those with severe OSAS. A similar trend was observed in the reduction of CMT, where the most notable improvements were seen in mild cases of OSAS compared to severe cases. This significant observation indicates that a condition causing fluctuations in blood oxygen levels can alter the function of retinal capillaries, thereby impacting the effectiveness of macular edema treatment.

In the mild OSAS group, one patient required only one intravitreal injection of anti-VEGF to treat their macular edema. In contrast, the other two patients in this group needed three injections to achieve similar results. In the moderate OSAS group, four patients received two anti-VEGF injections to reduce their CMT to less than 250 µm, while two other patients received three injections each. Meanwhile, patients in the severe OSAS group required one, two, or three intravitreal injections to achieve the same target CMT. These observations support the earlier findings that the severity of OSAS does not dictate improvements in CMT and visual acuity (Table 4).

**Table 4:** Number of injections needed in each group of OSAS OSAS, obstructive sleep apnea syndrome

Number of patients with OSAS as per severity	Number of injections needed		
	One	Two	Three
Mild (N = 3)	1 (33.3%)	0	2 (66.7%)
Moderate (N = 6)	0	4 (66.7%)	2 (33.3%)
Severe (N = 3)	1 (33.3%)	1 (33.3%)	1 (33.3%)

Additionally, the severity of OSAS influences the recurrence of macular edema. Patients with moderate to severe OSAS needed more doses of anti-VEGF injections to attain a comparable reduction in CMT compared to those with milder forms of the condition. In the severe OSAS group of three patients, one required anti-VEGF treatment only once, the second required two injections, and the third needed three injections to resolve macular edema.

There was a significant reduction in mean SFCT among the study participants compared to normal age-matched controls, which is attributable to the presence of higher CMT in participants with macular edema and resolution of the same with anti-VEGF therapy. However, the differences between mild, moderate, and severe OSAS and the normal population were insignificant. There was a weak positive correlation between the AHI and both the cumulative mean time and the ODI, with the r-value increasing at each visit; however, this correlation was not statistically significant. Additionally, there was no significant correlation between T90 and CMT. These parameters indicate that although such measurements may be useful while managing the problem of OSAS per se, their role in the management and outcome of macular edema arising from diabetic retinopathy and retinal vein occlusion may be limited.

The following table summarizes all the parameters of the patients, including the presence of OSAS, macular edema, visual acuity through the visits, and CMT measured at each visit (Table 5).

**Table 5:** Parameters of individual patients CMT, central macular thickness; OSAS, obstructive sleep apnea syndrome

Patient	Cause of macular edema	Degree of OSAS	Number of injections needed	First visit		Second visit		Third visit	
				Visual acuity (logMAR)	CMT (µm)	Visual acuity (logMAR)	CMT (µm)	Visual acuity (logMAR)	CMT (µm)
1	Central retinal vein occlusion	Moderate	3	0.6	533	0.4	414	0.2	249
2	Central retinal vein occlusion	Moderate	2	1	906	0.8	410	0.2	270
3	Central retinal vein occlusion	Severe	2	0.4	575	0.3	495	0.2	294
4	Central retinal vein occlusion	Mild	3	1	890	0.5	486	0	353
5	Diabetic macular edema	Moderate	2	0.8	496	0.4	365	0.2	225
6	Diabetic macular edema	Mild	3	1.4	943	1	573	0.6	468
7	Branch retinal vein occlusion	Moderate	3	0.8	720	0.4	541	0.4	301
8	Diabetic macular edema	Moderate	2	0.8	503	0.6	393	0.2	287
9	Central retinal vein occlusion	Mild	1	0.6	586	0.2	248	0	254
10	Branch retinal vein occlusion	Severe	1	0.8	606	0.4	276	0.2	269
11	Diabetic macular edema	Moderate	2	0.6	511	0.2	362	0.2	192
12	Diabetic macular edema	severe	3	1	663	0.8	573	0.6	468

## Discussion

The participants in our study were treated with anti-VEGF injections of ranibizumab and followed up as per the study protocol. Visual acuity, CMT, and SFCT were noted in all 12 participants across three follow-up visits. During this, intravitreal injections of ranibizumab were administered to evaluate the therapeutic response to anti-VEGF therapy and its correlation with OSAS. All participants underwent a level 1 PSG study before initiating anti-VEGF therapy for macular edema to diagnose the severity of OSAS.

Demographically, most participants were over 50 years of age, with a mean age of 51.33 ± 14.16 years; 50% of participants fell within the age range of 38-57 years. The cohort was predominantly male, comprising 75% of the total, while 25% were female. This skewed gender distribution could be due to various social and cultural attributes of Indian society, where the predominant workforce constitutes males. This could have resulted in more men, compared to women, actively seeking healthcare. The age distribution correlated with the prevalence of diabetes and hypertension since the common causes of macular edema in the young population were not studied in this study.

Our analysis revealed that the change in CMT was statistically significant across all severity grades of OSAS, with a p-value < 0.005, suggesting that the severity of OSAS does not influence the CMT. This finding corroborates the results of Kaba et al., who also reported no correlation between OSAS severity and macular edema in a cohort of diabetic retinopathy patients [5]. West and Turnbull found that while moderate to severe OSAS was prevalent in 54% of patients with clinically significant macular edema, they observed no significant relationship between the severity of OSAS and macular edema [6]. Zhang et al. conducted a large-scale study involving 880 hospitalized patients with type 2 diabetes, revealing no significant association between OSAS severity and diabetic retinopathy [7]. Notably, the literature review did not identify any published data that specifically studied the association between OSAS severity and macular edema in cases of retinal vein occlusion. This becomes important since retinal vein occlusion is the next most important cause of macular edema after diabetic retinopathy in terms of sheer numbers and disease burden.

It thus becomes apparent that the diagnosis of OSAS, coexisting with macular edema associated with diabetic retinopathy and retinal vein occlusion, is of greater importance in therapeutic management and holds prognostic value. The grading of severity of OSAS may not be of clinical importance in managing macular edema using ranibizumab as anti-VEGF therapy. This study also sheds light on the clinical outcome of macular edema secondary to retinal vein occlusions related to the presence of OSAS, which has not been studied before. The outcomes do not appear to differ regarding disease severity and therapeutic outcome, irrespective of the etiology of macular edema.

Conversely, a retrospective cohort study by Chiang et al. reported a different outcome, where severe OSAS was linked to a heightened incidence of refractory diabetic macular edema (27%) compared to non-severe OSAS (0%) [8]. Chang et al. found an association between severe OSAS and diabetic macular edema and PDR in a study of 317 patients [9]. We excluded PDR in our study since it portrays a very different spectrum of diabetic retinopathy where ongoing ischemia has formed new vessels with or without tractional membranes. The vitreoretinal interface is irreversibly altered, and it may be confounding to study the underlying factors influencing macular edema in the setting of PDR. Also, most cases of PDR in our setting are sufficiently advanced to warrant pan-retinal photocoagulation or vitreo-retinal surgery, which would permanently alter the oxygen demand of the retina and intraocular cytokine levels.

In our study, we noted a significant improvement in visual acuity when comparing the first and third visits in patients with mild to moderate OSAS. However, in severe OSAS cases, the visual acuity change following anti-VEGF injections was minimal and not statistically significant. This discrepancy may be explained by the fact that two-thirds of patients in the mild-moderate OSAS group presented with retinal vein occlusion accompanied by massive macular edema, leading to poorer baseline visual acuity. Additionally, it is important to recognize that presenting visual acuity is influenced by several disease and patient-specific factors not accounted for in our study, such as the chronicity of macular edema, systemic glycemic control, and the duration and severity of systemic comorbidities. Pelosini et al. demonstrated that the integrity of the IS-OS junction significantly predicts visual acuity, a factor not considered in our analysis [10]. However, we did not enroll participants with chronic atrophic cyst-like spaces on OCT scans and macular edema refractory to anti-VEGF therapy. Furthermore, Wakabayashi et al.’s study highlighted that better best-corrected visual acuity was associated with a larger area of foveal vascular perfusion in cases of branch retinal vein occlusion [11]. In our study, we did not assess retinal ischemia, which would require quantification of retinal perfusion of the fluorescein angiogram of retinal vasculature.

The findings in our study also indicated no significant correlation between the number of intravitreal anti-VEGF injections required to achieve a normal foveal contour and the severity of OSAS. Kaba et al.’s prospective cohort study noted that OSAS may diminish the efficacy of intravitreal anti-VEGF treatment due to higher serum levels of VEGF observed in patients with diabetic retinopathy and concurrent OSAS, implying a multifactorial influence [5]. Currently, there exists a paucity of literature comparing the severity of OSAS with the efficacy and frequency of intravitreal anti-VEGF treatments for clinically significant macular edema. However, this may be an important study area to understand the factors affecting the efficacy and frequency of anti-VEGF injections. It also becomes important because repeated anti-VEGF injections pose the greatest cost burden on the healthcare systems across the world, including higher clinic visits and interventions that require hospitalization, which becomes especially relevant in Indian settings, where intravitreal injections are given in the operating room as a standard practice.

In assessing SFCT, a significant reduction was observed in our study participants relative to a normal age-matched control group. However, no statistically significant differences in mean SFCT were noted across varying severity levels of OSAS compared to the population normative database. This contrasts with findings by Kara et al., who reported a thinner SFCT in patients with moderate to severe OSAS relative to normal controls [12]. A meta-analysis by He et al. affirmed that SFCT was significantly reduced in OSAS patients compared to normal individuals [13]. We additionally studied this factor since pachychoroid spectrum disorders (PSDs) are an established set of disease processes in the retina that alter the etiopathogenesis of the disease in question while also impacting therapeutic outcomes. With the data available from our study, macular edema associated with diabetic retinopathy and retinal vein occlusion does not appear to fall under the PSD group of disorders.

Our study has limitations. Notably, the small sample size and the follow-up duration were limited to three months. Some patients were lost to follow-up upon achieving good initial visual acuity improvements. Furthermore, a range of confounding variables were not controlled for, including age, the specific systemic conditions leading to macular edema, the status of macular perfusion, chronicity of macular edema, and the number of previous intravitreal injections. However, this was attempted to be addressed by excluding refractory macular edema based on previous clinical records at presentation, large atrophic spaces on OCT scans, and disruptions in the ellipsoid zone.

## Conclusions

This study aimed to evaluate the impact of OSAS on the severity of macular edema and the treatment response to intravitreal anti-VEGF therapy using ranibizumab in patients with diabetic retinopathy and retinal vein occlusion. The findings indicated no significant association between OSAS severity and CMT. However, there was a statistically significant improvement in visual acuity following anti-VEGF injections in patients with mild to moderate OSAS, whereas severe OSAS cases showed minimal, statistically insignificant improvement. It could therefore be deduced that OSAS’s severity could have some bearing on the therapeutic response in macular edema. Additionally, the study found a significant reduction in mean SFCT in the study population compared to the age-matched normative database. However, the differences were not statistically significant when comparing the different severities of OSAS individually. Further studies on a larger cohort may provide a greater understanding of the relation between OSAS and choroidal thickness associated with macular edema.

The study highlights the need for further prospective research with larger cohorts to better understand the effects of varying degrees of OSAS on macular edema and choroidal thickness and whether managing OSAS will help in the management of macular edema in terms of better reduction in macular thickness per injection and overall drop in the number of injections required to achieve normal CMT. Correlation with individual sleep study parameters did not appear to have a clinical impact on the outcomes of our study. Given that OSAS is a multisystem disorder associated with metabolic dysregulation, endothelial dysfunction, and systemic inflammation, a more profound understanding of its underlying mechanisms could enhance our knowledge of the causes of macular edema and device individualized interventions.
